# The persistence of stress-induced physical inactivity in rats: an investigation of central monoamine neurotransmitters and skeletal muscle oxidative stress

**DOI:** 10.3389/fnbeh.2023.1169151

**Published:** 2023-05-16

**Authors:** Trevor J. Buhr, Carter H. Reed, Olivia M. Wee, Ji Heun Lee, Li-Lian Yuan, Monika Fleshner, Rudy J. Valentine, Peter J. Clark

**Affiliations:** ^1^Department of Food Science and Human Nutrition, Iowa State University, Ames, IA, United States; ^2^Interdepartmental Neuroscience Program, Iowa State University, Ames, IA, United States; ^3^Department of Kinesiology, Iowa State University, Ames, IA, United States; ^4^Physiology and Pharmacology, Des Moines University, Des Moines, IA, United States; ^5^Department of Integrative Physiology, University of Colorado, Boulder, Boulder, CO, United States

**Keywords:** exercise motivation, monoamine neurotransmitter, adverse experience, stress physiology, health behavior adherence, histamine

## Abstract

**Introduction:**

Sedentary lifestyles have reached epidemic proportions world-wide. A growing body of literature suggests that exposures to adverse experiences (e.g., psychological traumas) are a significant risk factor for the development of physically inactive lifestyles. However, the biological mechanisms linking prior stress exposure and persistent deficits in physical activity engagement remains poorly understood.

**Methods:**

The purpose of this study was twofold. First, to identify acute stress intensity thresholds that elicit long-term wheel running deficits in rats. To that end, young adult male rats were exposed to a single episode of 0, 50, or 100 uncontrollable tail shocks and then given free access to running wheels for 9 weeks. Second, to identify stress-induced changes to central monoamine neurotransmitters and peripheral muscle physiology that may be maladaptive to exercise output. For this study, rats were either exposed to a single episode of uncontrollable tail shocks (stress) or left undisturbed in home cages (unstressed). Eight days later, monoamine-related neurochemicals were quantified by ultra-high performance liquid chromatography (UHPLC) across brain reward, motor, and emotion structures immediately following a bout of graded treadmill exercise controlled for duration and intensity. Additionally, protein markers of oxidative stress, inflammation, and metabolic activity were assessed in the gastrocnemius muscle by Western blot.

**Results:**

For experiment 1, stress exposure caused a shock number-dependent two to fourfold decrease in wheel running distance across the entire duration of the study. For experiment 2, stress exposure curbed an exercise-induced increase of dopamine (DA) turnover measures in the prefrontal cortex and hippocampus, and augmented serotonin (5HT) turnover in the hypothalamus and remaining cortical area. However, stress exposure also caused several monoaminergic changes independent of exercise that could underlie impaired motivation for physical activity, including a mild dopamine deficiency in the striatal area. Finally, stress potently increased HSP70 and lowered SOD2 protein concentrations in the gastrocnemius muscle, which may indicate prolonged oxidative stress.

**Discussion:**

These data support some of the possible central and peripheral mechanisms by which exposure to adverse experiences may chronically impair physical activity engagement.

## 1. Introduction

The financial burdens and premature mortality rates resulting from sedentary lifestyles are related to the development of the most debilitating chronic diseases and mental health disorders, including diabetes, obesity, heart disease, cancers, drug abuse, anxiety, and depression, to name some examples (Booth et al., 2008; Bauer et al., 2014; Gonzalez et al., 2017; Brellenthin and Lee, 2018; Park et al., 2020). Identifying the central and peripheral biological factors that mediate a decreased willingness to be active may be critical to the treatment and prevention of the sedentary lifestyle, especially within populations that have no apparent underlying disability preventing physical activity engagement. Mounting evidence suggests that exposure to adverse events (e.g., psychological traumas) may contribute to the development of sedentary lifestyles (Moraska and Fleshner, 2001; Stults-Kolehmainen and Sinha, 2014; Vasquez et al., 2016; DeVallance et al., 2017; van den Berk-Clark et al., 2018; Teychenne et al., 2019; Jiang et al., 2020; Raney et al., 2022), providing a possible factor linking psychological traumas and increased risks for chronic disease, substance use, and mood and anxiety disorders. However, relatively little is known about how stress exposure can alter physiology in manners that make individuals more prone to chronic inactivity (Boyington et al., 2015), especially long after the stressor is no longer present. A better understanding of stress-induced physiological changes that contribute to persistent physical inactivity could be central to the discovery of effective approaches that prevent or reverse sedentary lifestyles.

Rodent models represent a useful tool to address the consequences of acute stress on persistent physical inactivity, as they display a stress-response and neuroanatomy similar to humans, but also allow for a mechanistic approach to studying the brain that is not readily possible in humans. A previous study has shown that young adult rats exposed to a single episode of uncontrollable tail shocks (i.e., acute stress) develop a long-term reduction in physical activity, as revealed by a potent decrease in daily running distance on wheels compared to non-stressed rats that persists for at least months (Moraska and Fleshner, 2001). This is particularly interesting because wheel running is naturally rewarding to rodents, as rodents will spontaneously run great distances on wheels (e.g., ∼3–16 km/day), work to obtain access to running wheels, develop a conditioned preference to contexts associated with wheel access, and even run on wheels placed in the wild (Iversen, 1993; Novak et al., 2012; Meijer and Robbers, 2014; Herrera et al., 2016; Medrano et al., 2021). Furthermore, depression-like deficits in rat-motivated behaviors, including preference for sweet substances, food consumption, goal-directed spatial learning, social interaction, and exploration of new contexts recover to non-stressed levels in approximately 72 h post exposure to the uncontrollable tail shock paradigm (Desan et al., 1988; Warren et al., 1991; Will et al., 1998; Maier and Watkins, 2005; Christianson et al., 2008). Tasks requiring rigorous forced locomotor activity, like 15 min of forced swim test and approximately an hour of shuttle box escape testing also recover to non-stressed levels within the same period (Weiss et al., 1981; Desan et al., 1988; Warren et al., 1991; Maier and Watkins, 2005; Strong et al., 2011). Taken together, these data suggest the development of mammalian sedentary behavior following exposure to acute stress may not be just a simple correlate of mood or anxiety disorders, but instead unique to changes within central and/or peripheral biological substrates that chronically deter the willingness to engage in voluntary physically exertive activities. Identifying the biological consequences of acute stress on long-term physical activity deficits could begin to elucidate the internal processes that contribute to the development of sedentary lifestyles.

Studies on variations in physical activity levels in humans and rodent models have primarily focused on differences in genetic, anatomical, and physiological functions in the brain and muscle (Lightfoot et al., 2018). Exposure to stressors of sufficient length or intensity can lead to maladaptive physiological changes in these tissue sources, which over a lifetime, result in a metabolically demanding dysregulation of pro-inflammatory, oxidative stress, and hormonal processes hypothesized to give rise to unhealthy lifestyle practices (Schiavone et al., 2013; Slavich and Irwin, 2014; Lacey et al., 2020; Tay and Lim, 2020), such as physical inactivity. Moreover, an accumulating body of literature suggests that abnormalities in genetic and physiological modulators of brain monoamine neurotransmitter activity underlie differences in human and rodent exercise motivation (Rhodes and Garland, 2003; Good et al., 2015; Claghorn et al., 2016; Saul et al., 2017; Greenwood, 2019; Tanner et al., 2022). For instance, variations in genes for nescient helix-loop-helix 2 (NHLH2) and monoamine-oxidase-A (MAO-A) that can regulate monoamine levels have been argued to result in hypo-dopaminergic or -serotonergic states in the brain that may reduce habitual exercise engagement (Good et al., 2015; Greenwood, 2019). Furthermore, increases in the ratio of serotonin (5HT) to dopamine (DA) activity across reward, motor, and emotion structures are associated with reduced exercise motivation and output (Meeusen et al., 2006). Therefore, central or peripheral oxidative stress and pro-inflammatory events that can arise from exposure to adverse events may disrupt monoamine activity in the brain that regulates physical activity engagement. However, the central and peripheral factors that contribute to persistent deficits in physical activity following adverse experiences are currently unknown.

The purpose of this study was twofold. First, to identify acute stress intensity thresholds that elicit long-term wheel running deficits in rats. A prior study showed that exposing rats to a single episode of 100 uncontrollable tail shocks is sufficient to produce a robust deficit in wheel running behavior that persists months beyond anxiety- and depression-like behaviors (Weiss et al., 1981; Desan et al., 1988; Warren et al., 1991; Moraska and Fleshner, 2001; Maier and Watkins, 2005; Christianson et al., 2008). However, the influence of changes to shock number on physical activity levels is unknown, yet could provide insights into the possible conditions necessary to develop persistent aversions for physical activity. Second, to identify possible stress-induced changes to central monoamine neurotransmitters and peripheral muscle physiology that may be maladaptive to physical exertion during exercise. To that end, monoamine-related neurochemicals were quantified across brain reward, motor, and emotion structures of stressed and unstressed rats immediately following a bout of treadmill exercise that was controlled for duration and intensity. Stress-altered monoaminergic responses to a controlled bout of exercise could provide insight into the neural mechanism by which adverse experience persistently disrupts physical activity levels. In addition, the gastrocnemius muscle was extracted in the same rats to measure biomarkers of oxidative stress, inflammatory, and metabolic factors that could underlie peripheral contributions to stress-induced physical activity deficits. Indeed, very little work has been completed to understand the impact of adverse experiences on skeletal muscle physiology outside of heat stress, especially in the context of physical activity engagement. The results of this study provide some preliminary insight into the possible relationships between stressed-induced central and peripheral changes that could be contributing to long-term reductions in physical activity levels.

## 2. Materials and methods

### 2.1. Animals

Adult male Sprague Dawley (SD) rats (220–250 g upon arrival) were chosen per previous work documenting long-term wheel running deficits that persist for at least months (Moraska and Fleshner, 2001). The first cohort of rats (*n* = 18) was used to characterize wheel running volumes following exposure to a single episode of 0, 50, or 100 tail shocks. The second cohort (*n* = 26) was used to assess differences in performance between stressed and non-stressed rats on a bout of graded treadmill exercise. The third cohort of rats (*n* = 36) was used to identify stress-induced changes to brain monoaminergic responses during running, as well as hind limb muscle oxidative stress, inflammatory, and metabolic factors that could underlie persistent deficits in physical activity output. The experimental parameters of each set of rats is described in detail in the sections below. Food (ENVIGO, Tekland 2014, g/kg diet: crude protein, 143; fat, 40; carbohydrate, 480; crude fiber, 41; neutral detergent fiber, 180; ash, 47; AIN-93 mineral and vitamin mix) and water were provided *ad libitum*. Sample sizes were determined by power analyses completed on our previous work (Bauer et al., 2021; Buhr et al., 2021; Reed et al., 2021). Rooms were controlled for temperature (21 ± 1°C) and photo-period (12:12 L:D, “Lights-Off” at 6 PM) for the entire study. All procedures were approved by the Iowa State University Institutional Animal Care and Use Committee and adhered to NIH guidelines.

### 2.2. Experiment 1: stressor intensity and physical activity deficits

The purpose of this experiment was to examine the influence of differences in stress intensity on long-term physical activity engagement. While previous work has demonstrated that SD rats exposed to an episode of 100 uncontrollable tail shocks demonstrate a robust and prolonged deficit in running wheel activity (Moraska and Fleshner, 2001), the influence of exposure to fewer shocks on long-term wheel running engagement is unknown. Therefore, upon arrival, rats were individually housed in a standard laboratory cage with locked 13-inch diameter running wheels (STARR Life Sciences) for 1 week. Rats were then randomly assigned to receive a single episode of 0, 50, or 100 (*n* = 6/shock number group) uncontrollable tail shocks (described below). Forty-eight hours later, running wheels were unlocked and rats were allowed to run freely for the 9-week duration of the study. The 48 h delay between tail shocks and wheel access was done to minimize the possible development of an association between tail shock exposure and free access to running wheels. Wheel running distance was collected every hour for 9 weeks using Mini-Mitter software (STARR Life Sciences). Average daily running distance for each of the 9-weeks, and diurnal rhythms during weeks 2, 6, and 8 were recorded.

#### 2.2.1. Uncontrollable stress

Rats were assigned to either receive uncontrollable tail shocks (Stress) or left undisturbed in their home cages (No stress). The tail shock paradigm followed our previous publications (Clark et al., 2014, 2015; Reed et al., 2021). Approximately 2–5 h into the light cycle, rats that received tail shocks were restrained in flat bottom Plexiglas tubes with the tail protruding from the back where electrodes were placed to deliver 50 or 100, 1.25 mA current, 5-s tail shocks on a variable 1 min inter-shock interval. Thus, rats that received 50 tail shocks were on restrained for half of the total time of rats exposed to 100 shocks. Rats that did not receive stress (i.e., 0 shocks) remained undisturbed in home cages in a different room during the tail shocks.

### 2.3. Experiment 2: stress-altered central monoamine and peripheral muscle factors during exercise

#### 2.3.1. Treadmill paradigm

The purpose of this pilot experiment was to identify the point at which stressed and unstressed rats reach failure during a controlled bout of exercise. This was completed with two separate cohorts of rats that differed in treadmill habituation protocols. Upon arrival, rats were individually housed in standard laboratory cages for 1 week.

In the *first cohort*, rats underwent a three consecutive day treadmill training procedure. Each day groups of 8 rats were placed onto an 8-lane treadmill (constant grade of 15°), 3 h into the dark cycle, for 5 min to acclimate to being moved to a new location. During the first 2 days of treadmill training, after 5 min of acclimation, the treadmill was turned on for 5 min at a speed of 10 m/min before animals were returned to their home cages. During the final day of treadmill training, the treadmill was turned off for 2.5 min after the first 5 min bout of running at 10 m/min as a “rest” period. After the “rest” period, the treadmill was turned back on at a speed of 10 m/min for a final 5 min before the animals were returned to their home cages. Animals were prodded forward with a plastic tongue depressor if the animal rested hind limbs on the back wall of the treadmill lane or were picked up and turned around if resting forelimbs on the back wall of the treadmill lane. Forty-eight hours later, rats were randomly assigned to receive 0 (no stress, *n* = 5) or 100 uncontrollable tail shocks (stress, *n* = 5) as previously described.

Within 5 days following stress exposure, rats completed a single bout of graded-intensity treadmill exercise until failure. During this trial, rats began at a speed of 10 m/min which was increased by 1 m/min every 2 min until failure to continue running was reached, as defined by three episodes of immobility that lasted for at least 2 s. Animals were encouraged to continue to run with a plastic tongue depressor that was gently pressed against the base of their tail and body when immobile. This approach was chosen to encourage running while minimizing stress exposure by avoiding electrical shock or bursts of air to motivate rodents. The time until the rats reached final failure was recorded.

Rats in cohort 2 underwent a five consecutive day treadmill training procedure that followed the same parameters as cohorts 1, except that the animals received two additional days of treadmill training before and another “re-acclimation” episode after exposure to tail shock. For these additional habituation days, the treadmill was turned off for 2.5 min after the first 5 min bout of running at 10 m/min as a “rest” period. After the “rest” period, the treadmill was turned back on at a speed of 10 m/min for a final 5 min before the animals were returned to their home cages. Forty-eight hours later, rats were randomly assigned to receive 0 (no stress, *n* = 8) or 100 uncontrollable tail shocks (stress, *n* = 8) as previously described. A “re-acclimation” trial occurred 6 days after stress exposure, in which animals were placed on the treadmill for 2.5 min while the treadmill was off, followed by the treadmill being turned on at 10 m/min for 5 min. Eight days following stress exposure (48 h after “re-acclimation”), rats completed a single bout of graded intensity treadmill exercise until failure following the same parameters described in cohort 1. Eight days was chosen because it is sufficiently beyond the 72 h period during which anxiety- and depression-like behaviors are observed following exposure to uncontrollable tail shock.

#### 2.3.2. Treadmill paradigm for investigating brain neurochemical and muscle factors

A follow-up study was completed to examine the possible influence of acute stress on changes to central monoamines and skeletal muscle factors that could contribute to reduced exercise output. Rats were randomly assigned to treadmill running or control conditions. Rats in the running conditions were acclimated to treadmills exactly as described in cohort 2 of the previous section. Rats in the control condition were placed on the treadmill for the same period of time as the running group, but the treadmills remained off. Forty-eight hours later, rats in both running and control conditions were randomly assigned to receive 0 (no stress; *n* = 9 control, *n* = 9 run) or 100 uncontrollable tail shocks (stress; *n* = 9 control, *n* = 9 run). Eight days following stress exposure, rats in the running condition completed a single bout of vigorous treadmill exercise, which followed the exact parameters in experiment 2, but ended at after 15 min. Fifteen min was chosen because this period was just minutes before the second cohort of stressed rats reached failure on the previously described treadmill paradigm, thus serving as a set period of physical activity whereby physiological processes driving reduced motivation to continue exercise could become captured in stressed compared to unstressed rats. Rats in the control condition were placed on the treadmill that was left turned “off” for 15 min. Thus, rats in the control group were exposed to the treadmill environment for the same duration as running animals, but without receiving the physically exertive activity. Immediately following the 15 min period on treadmills, rats were euthanized by rapid decapitation without anesthesia, and brains and gastrocnemius muscle were quickly extracted. Only rats that completed the entire 15 min treadmill episode without reaching failure were included for analysis.

#### 2.3.3. High performance liquid chromatography and analysis

Brain regions containing the prefrontal cortex, remaining caudal cortical area, hypothalamus, cerebellum, striatum, hippocampus, and brainstem areas were rapidly microdissected on a glass plate placed over ice [for detailed methods see our previous publications (Bauer et al., 2021; Buhr et al., 2021; Reed et al., 2021)]. These brain regions were chosen because they are components of motor, emotion, and reward pathways that could contribute to exercise motivation. Microdissected brain areas were placed in pre-weighed cryovials containing 0.2M perchloric acid, flash-frozen with liquid nitrogen, weighed again to obtain sample weights, and then stored at −80°C in an Ultra-Low freezer until Ultra-High Performance Liquid Chromatography with electrochemical detection (UHPLC) processing.

Detailed UHPLC methodology for assessing monoamine-related neurochemicals can be found in our previous publications (Bauer et al., 2021; Buhr et al., 2021). Neurochemical measurements obtained from UHPLC were corrected for by gram weight of frozen tissue in each rat brain region (i.e., mg of neurochemical/gram of tissue) for dopamine (DA), homovanillic acid (HVA), 3,4-dihydroxyphenylacetic acid (DOPAC), serotonin (5HT), 5-hydroxyindoleacetic acid (5HIAA), and norepinephrine (NE). A large unidentified peak obscured our ability to obtain accurate measurements dimethylhexylamine (DMHA), therefore this NE metabolite was not included in the results. Note that any reported changes to NE levels without a corresponding measurement of DMHA are difficult to interpret. Moreover, levodopa (L-Dopa), Histamine (HIS), and Epinephrine (EPI) concentrations are reported in regions that reliable measurements could be obtained, as very little is known about how a bout of exercise can influence these neurochemicals across the brain.

The ratio of 5HT and DA metabolite to respective neurotransmitter concentration [i.e., 5-HIAA/5-HT and (HVA + DOPAC)/DA] was calculated as a measure of neurotransmitter turnover in each sample condition (Nissbrandt and Carlsson, 1987; Phillips et al., 1989). Moreover, a shift in the ratio favoring 5HT:DA within the brain has been previously reported to contribute to reduced exercise motivation and fatigue (Meeusen et al., 2006). Therefore, the ratio of 5HT:DA Turnover (i.e., 5HIAA/5HT:HVA + DOPAC/DA) was also considered within each brain region.

#### 2.3.4. Western blot analysis

Gastrocnemius muscle was extracted and flash-frozen in cryovials with liquid nitrogen and stored at −80°C in an Ultra-Low freezer until Western blot analysis. Roughly 50 mg of muscle was homogenized in ice cold RIPA buffer at a 1:10 ratio of tissue to solution. Phosphatase and protease inhibitors were combined with the RIPA buffer for homogenization (#P0044, Sigma-Aldrich, Burlington, MA; and #78425, Thermo Fisher Scientific, Waltham, MA, respectively). After homogenization sample protein was extracted and protein concentration was quantified using the Pierce BCA Protein Assay (#23225, Thermo Fisher Scientific, Waltham, MA). Next, equal concentrations of protein were loaded into 4–15 or 4–20% gradient Stain-Free Criterion gels (#567085 and 5678095, respectively, Bio-Rad, Hercules, CA) and separated via electrophoresis. Following electrophoresis, gels were activated with UV light and then transferred to polyvinylidene (PVDF) membranes (#IPVH00005, Millipore Sigma, Burlington, MA). Total lane protein was imaged following transfer and membranes were then blocked using 5% non-fat milk combined with tris-buffered saline plus 0.1% Tween-20 (TBST) (#9005-64-5, Thermo Fisher Scientific, Waltham, MA). Once blocking was completed, membranes were washed in TBST and incubated overnight in primary antibody. After incubation in primary antibody membranes were washed in TBST and incubated for 1 h at room temperature in HRP-conjugated secondary antibody. Secondary antibodies ranged from 1:1,000–1:2,000 dilutions. Following incubation, membranes were washed in TBST and incubated with either SuperSignal West Pico Plus Chemiluminescent Substrate (#34580 Thermo Fisher Scientific, Waltham, MA) or SuperSignal ELISA Femto Maximum Sensitivity Substrate (Thermo Fisher Scientific, Waltham, MA). Images were taken using the ChemiDoc XRS Imaging System (Bio-Rad, Hercules, CA) and protein densitometry was performed with Image Lab 6.0.1 software from Bio-Rad. Target proteins were normalized to total lane instead of common housekeeping proteins (e.g., GAPDH or β-actin). Normalizing for total lane protein accounts for the intensity of all proteins loaded in a lane, and variation that can occur during electrophoresis and transfer (Taylor and Posch, 2014). This method has been validated against traditional normalization tools, such as use of housekeeping proteins (Colella et al., 2012; Gurtler et al., 2013; Li and Shen, 2013), while eliminating the possible confounds that arise when housekeeping proteins change due to the experimental conditions (Ferguson et al., 2005).

Oxidative stress, inflammation, and energy balance in muscle have been shown to interact in complex and coordinated manners to influence exercise behavior. Therefore, the following markers were measured in gastrocnemius muscle. (1) Phosphorylated-AMPK ^*T*172^ and total AMPK (cat. # 2531 and 2532, Cell Signaling Technology) were used to investigate possible differences in factors that regulate energy metabolism during treadmill running (Park et al., 2002). (2) SIRT1 protein (#07-131, Millipore) was assessed due to its involvement in energy metabolism from glucose homeostasis (Suwa et al., 2008), which could become altered during exercise following stress. Heat shock proteins (HSP) can become upregulated in response to oxidative stress and inflammatory responses that follow episodes of stress and exercise (Tupling, 2008; Li et al., 2011). Therefore, (3) HSP70 (cat. # sc-24, Santa Cruz Biotechnology), (4) total and phosphorylated HSP27 (cat. # sc-13132 and sc-166693, Santa Cruz Biotechnology), and (5) HSP90 (4874, Cell Signaling Technology), as well as (6) Heat Shock Factor 1 (HSF1; cat. #sc-17757, Santa Cruz Biotechnology) were analyzed. Moreover, protein concentrations of (7) SOD1 (cat. #sc-515404, Santa Cruz Biotechnology) and (8) SOD2 (cat. #sc-133134, Santa Cruz Biotechnology) were considered as they can also be indicative of oxidative stress (Franco et al., 1999; Hollander et al., 2001). (9) IL-1β (cat # sc-515598, Santa Cruz Biotechnology) is a key mediator of the inflammatory response to stressors and plays a role in fatigue responses to exercise (Carmichael et al., 2006). (10) TNFα (cat. # sc-52746, Santa Cruz Biotechnology) can act as a proinflammatory cytokine at the acute phase of stress responses and during fatigue (Weinstock et al., 1997; Moldoveanu et al., 2000). (11) IL-6 (cat. # sc-57315, Santa Cruz Biotechnology) is an immune factor that may serve as an interface between muscle contraction and changes in energy metabolism during exercise, and therefore may contribute to exercise output (Pedersen et al., 2001).

### 2.4. Statistical analysis

For experiment 1, average daily running distance for each week was compared between groups of rats that received 0, 50, or 100 tail shocks by a repeated measures ANOVA. For experiment 2, time to failure on the treadmill task for non-stressed and stressed rats was compared by *t*-test. Brain region neurotransmitter concentration and turnover measures, as well as western blot muscle protein concentrations were analyzed by two-way ANOVA, with stress (0 or 100 tail shocks) and exercise (control or treadmill conditions) as factors. *Post hoc t*-tests with Tukey corrections for multiple comparisons were completed on ANOVAs with statistically significant interactions between factors or both main effects of stress and exercise. Neurochemical concentration data points that were greater than two standard deviations from the mean were excluded from statistical analysis, as reported in the degrees of freedom. Statistical significance level was set to 0.05. ANOVAs with statistically non-significant trends were reported up to 0.10 in the text, but were not noted in the figures.

## 3. Results

### 3.1. Experiment 1: stressor intensity and physical activity deficits

#### 3.1.1. Average daily running distance

**FIGURE 1:**
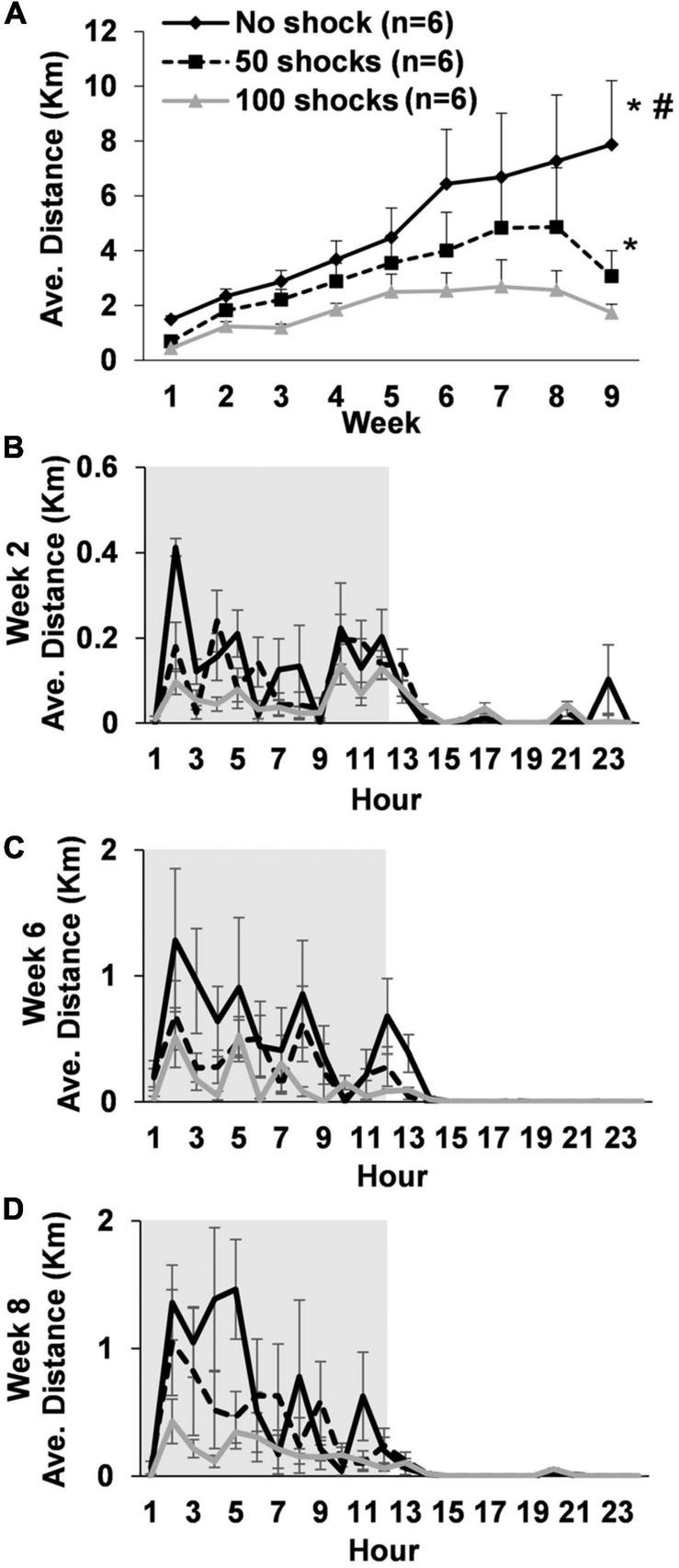
Long-term deficits in wheel running develop following exposure to acute stress. **(A)** Young adult male rats exposed to a single episode of uncontrollable tail shocks display deficits in daily wheel running distance that persisted for at least 9 weeks. Stressed and non-stressed rats display normal diurnal rhythms (animal colony “lights-off” period in gray background) during weeks **(B)** 2, **(C)** 6, and **(D)** 8. However, overall distance traveled on running wheels each hour decreased proportionally to the greater number of tail shocks that rats received during stress. *P < 0.05 from no shock, ^#^*P* < 0.05 from 50 shocks.

Rats gradually increased average daily running distances over the 9 weeks [*F*(8,119) = 5.24, *P* < 0.0001]. However, rats that received stress displayed a two to fourfold decrease in average daily running distance across weeks that was dependent upon the number of tail shocks that they received [*F*(2,15) = 19.81, *P* < 0.0001] (Figure 1A). Indeed, *post hoc* analysis revealed that rats exposed to no tail shocks had an average daily running distance each week that was greater than rats exposed to 50 (*P* = 0.0438) or 100 tail shocks (*P* < 0.0001). Moreover, rats exposed to 50 tail shocks ran further daily each week than rats that received 100 tail shocks (*P* = 0.0277). Average weekly running distance across the entire study for each rat can be found in Supplementary Table 1.

#### 3.1.2. Diurnal rhythms

Average hourly wheel running distance was considered across days during weeks 2, 6, and 8 to determine if exposure to stress caused abnormalities to daily running patterns. Statistically significant stress by hour interactions were observed during weeks 2 [*F*(69,322) = 1.40, *P* = 0.0303] (Figure 1B), 6 [*F*(69,322) = 1.63, *P* = 0.0026] (Figure 1C), and 8 [*F*(69,322) = 1.87, *P* = 0.0002] (Figure 1D). Despite some relatively subtle variations in hourly running patterns between groups, rats in each stress condition overall displayed normal diurnal activity patterns. However, rats ran less overall at a given time point during the dark cycle in a manner that was dependent upon the number of shocks they received.

### 3.2. Experiment 2: stress-altered central monoamine and peripheral muscle factors during exercise

#### 3.2.1. Time to failure on treadmill

**FIGURE 2:**
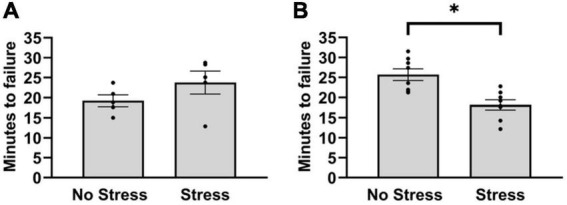
Latency to reach failure on a bout of graded treadmill exercise for non-stressed and stressed rats. **(A)** Stressed and non-stressed rats did not differ in running duration during a graded bout of treadmill exercise when rats received three habituation trials before exposure to tail shocks. **(B)** However, when rats received five habituation trails before and another post exposure to tail shocks, stressed rats ran for a shorter duration compared to non-stressed rats on a graded bout of treadmill exercise. Bars graphs are group mean (± SEM) with dots representing each animal’s latency. *P < 0.05 from Stress.

In the first cohort, stressed rats and non-stressed rats did not differ in duration to reach failure on the treadmill test [*T*(8) = 1.40, *P* = 0.20]. Since the treadmill outcomes after five rats per group did not differ in a magnitude and direction that is consistent with wheel running deficits, the number of habituation episodes was increased in a second cohort of rats. This was done to reduce the possible influence of stress on running duration during the treadmill test. In the second cohort, rats that were exposed to stress reached failure 31% faster during treadmill running than non-stressed rats [*T*(14) = 3.41, *P* = 0.0039] (Figure 2B). This resulted in an overall distance traveled of 405 m (±39) for non-stressed rats and 256 m (±31) for stressed rats. Moreover, non-stressed rats reached a final velocity of 16.0 m/min (±0.4) and stressed rats reached 14.0 m/min (±0.4). Since stressed rats took approximately 18 min to reach failure, the treadmill procedure was repeated in a separate cohort of stressed and non-stressed rats (see section “Materials and methods”) that were sampled after 15 min, chosen to investigate stress-related changes in central monoamine and skeletal muscle factors at a time point that rats were nearing failure. Outcomes of neurochemical analysis and muscle protein concentrations are described below.

#### 3.2.2. Neurochemical analysis

##### 3.2.2.1. Striatal area

**FIGURE 3:**
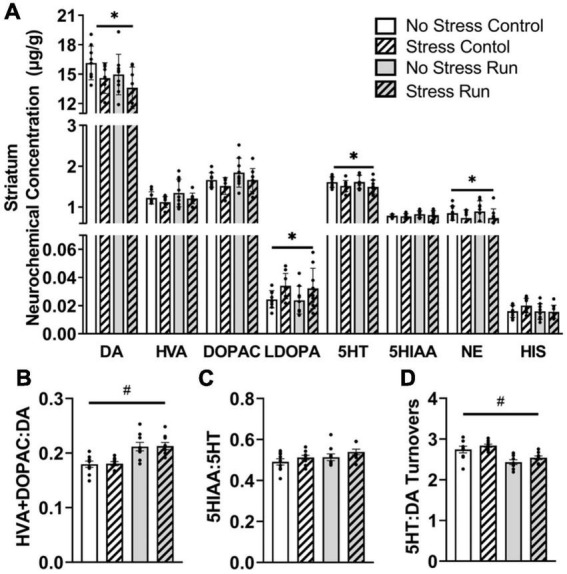
The interaction between stress and a bout of graded treadmill exercise on monoamine-related neurochemical profiles in the striatal area. **(A)** Rats exposed to stress had lower DA, 5HT, and NE concentrations, as well as higher L-dopa concentrations compared to non-stressed rats. **(B)** Treadmill running increased DA turnover measures. **(C)** Neither stress nor exercise influenced 5HT turnover measures. **(D)** Treadmill running lowered the ratio of 5HT:DA turnover measures in the striatum compared to non-running control rats. Bars graphs are group mean (± SEM) with dots representing each animal’s neurochemical concentration. *P < 0.05 main effect of stress, ^#^*P* < 0.05 main effect of exercise.

Rats exposed to stress had lower DA [*F*(1,32) = 5.28, *P* = 0.0281] and increased L-dopa concentrations [*F*(1,32) = 6.71, *P* = 0.0143] compared to non-stressed rats (Figure 3A). Moreover, stress exposure caused a statistically non-significant trend toward decreased HVA [*F*(1,32) = 2.90, *P* = 0.0985] and DOPAC [*F*(1,32) = 3.48, *P* = 0.0712] concentrations. Finally, treadmill exercise caused a statistically non-significant trend toward lowered DA [*F*(1,32) = 2.98, *P* = 0.094] and elevated DOPAC [*F*(1,32) = 3.54, *P* = 0.0692] concentrations, which contributed to a running related increase of DA turnover measures [*F*(1,32) = 28.09, *P* < 0.0001] compared to the control condition (Figure 3B).

Stress exposure also lowered 5HT concentrations in the striatum [*F*(1,32) = 4.43, *P* = 0.0432] without causing any notable changes to 5HIAA. Treadmill running resulted in a statistically non-significant trend toward a higher 5HT turnover measures compared to control conditions [*F*(1,32) = 3.12, *P* = 0.0867] (Figure 3C). Overall, the ratio of 5HT:DA turnover measures in the striatum were lowered by treadmill running compared to the control condition [*F*(1,32) = 27.31, *P* < 0.0001] (Figure 3D).

Finally, stress exposure lowered NE concentration in the striatal area [*F*(1,32) = 6.19, *P* = 0.0183].

##### 3.2.2.2. Prefrontal cortex

**FIGURE 4:**
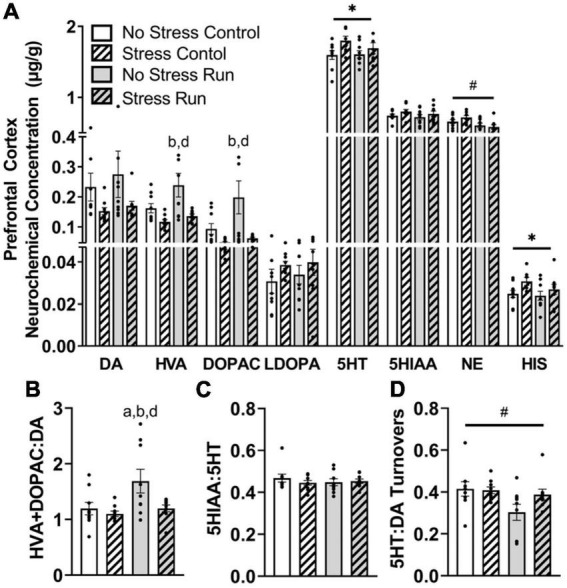
The influence of stress on monoamine-related neurochemical profiles in the prefrontal cortex following a graded bout of treadmill exercise. **(A)** Stress exposure blunted the increase of HVA and DOPAC concentrations following a graded bout of treadmill running, as well as augmented 5HT and HIS concentrations in the prefrontal cortex (PFC). Moreover, treadmill running lowered NE concentrations in the PFC. **(B)** Stress exposure prevented the treadmill running-induced elevation of DA turnover measures. **(C)** Neither stress nor running had a notable influence 5HT turnover measures. **(D)** Treadmill running lowered the ratio of 5HT:DA turnover. Bars graphs are group mean (± SEM) with dots representing each animal’s neurochemical concentration. *P < 0.05 main effect of stress, ^#^*P* < 0.05 main effect of exercise. ^a^*P* < 0.05 from No Stress Control, ^b^*P* < 0.05 from Stress Control, ^d^*P* < 0.05 from Stress Run.

Stress exposure blunted the enhancement of dopamine metabolite concentrations during treadmill running in the prefrontal cortex (Figure 4A). Indeed, HVA concentrations were augmented by treadmill running [*F*(1,32) = 4.94, *P* = 0.0334], but lowered by stress exposure [*F*(1,32) = 11.61, *P* = 0.0018]. *Post hoc* analysis revealed that rats in the No Stress Run group had greater HVA concentrations than rats in the Stress Control (*P* = 0.0020) and Stress Run (*P* = 0.0108) groups. DOPAC displayed a consistent trend, whereby the bout of treadmill running augmented [*F*(1,32) = 4.17, *P* = 0.0495] and stress exposure lowered [*F*(1,32) = 9.69, *P* = 0.0039] neurochemical concentrations. *Post hoc* analysis revealed that rats in the No Stress Run group had greater DOPAC concentrations than rats in the Stress Control (*P* = 0.0049) and Stress Run (*P* = 0.0116) groups. Moreover, stress exposure caused a statistically non-significant trend toward lower DA concentrations compared to no stress conditions [*F*(1,32) = 4.03, *P* = 0.0533]. A net increase of DA turnover was observed in the treadmill running compared to the control condition [*F*(1,32) = 5.44, *P* = 0.0261] that was lowered by stress exposure [*F*(1,32) = 5.29, *P* = 0.0282] (Figure 4B). *Post hoc* analysis revealed that rats in the No Stress Run group had a higher ratio of HVA + DOPAC:DA than all three groups; No Stress Control (*P* = 0.0450), Stress Control (*P* = 0.0129), and Stress Run (*P* = 0.0475).

Stress exposure increased 5HT concentrations in the prefrontal cortex [*F*(1,32) = 4.90, *P* = 0.0341] compared to the no stress condition, without having any notable effect on 5HIAA or 5HT turnover measures (Figure 4C). Treadmill running had no influence on any 5HT-related measures compared to the control condition. However, the ratio of 5HT:DA turnover was mildly reduced by treadmill running compared to control conditions [*F*(1,32) = 4.91, *P* = 0.0340] (Figure 4D).

Finally, treadmill running lowered NE [*F*(1,32) = 10.51, *P* = 0.0028], whereas stress elevated HIS [*F*(1,32) = 4.80, *P* = 0.0358] concentrations in the prefrontal cortex.

##### 3.2.2.3. Caudal cortical area

**FIGURE 5:**
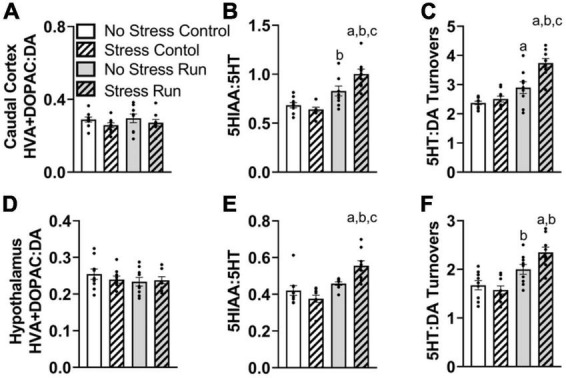
The interaction between stress and a bout of treadmill exercise on monoamine-related neurochemical profiles in the caudal cortical and hypothalamic areas. In the remaining cortical area (without PFC), **(A)** neither stress nor exercise influenced DA turnover measures. **(B)** Stress augmented the increase of 5HT turnover measures in the caudal cortex following a bout of treadmill running. **(C)** Stress also caused an augmentation of the ratio of 5HT:DA turnover in response to treadmill running. **(D)** In the hypothalamic area, neither stress nor exercise influenced DA turnover measures. **(E)** Stress exposure augmented 5HT turnovers measures in response to a bout of treadmill running. **(F)** Stress also augmented the ratio of 5HT:DA turnover in the hypothalamus following treadmill running. Bars graphs are group mean (± SEM) with dots representing each animal’s neurochemical concentration. ^a^*P* < 0.05 from No Stress Control, ^b^*P* < 0.05 from Stress Control, ^c^*P* < 0.05 from No Stress Run.

No dopamine-related measures were notably influenced by stress or treadmill running in the caudal cortical area (Supplementary Figure 1A and Figure 5A). There was a trend toward elevated 5HIAA concentrations by treadmill running that failed to reach statistical significance [*F*(1,32) = 2.92, *P* = 0.0974]. However, there was an interaction between treadmill running and stress conditions for 5HT turnover measures [*F*(1,32) = 7.50, *P* = 0.0100] (Figure 5B). *Post hoc* analysis revealed that the Stress Run group had a greater ratio of 5HIAA:5HT than No Stress Control (*P* < 0.0001), Stress Control (*P* < 0.0001), and No Stress Run (*P* = 0.0199) groups. No Stress Run group also had a greater 5HT turnover than the Stress Control group (*P* = 0.0480).

Since DA turnover measures did not differ across groups, a similar interaction was observed between exercise and stress conditions for 5HT:DA turnovers [*F*(1,32) = 6.50, *P* = 0.0158] (Figure 5C), whereby *post hoc* analysis indicated that the Stress Run group had a greater ratio of 5HT:DA turnover than No Stress Control (*P* < 0.0001), Stress Control (*P* < 0.0001), and No Stress Run (*P* = 0.0009) groups. Moreover, the No Stress Run group also had a greater 5HT:DA turnover ratio than the No Stress Control group (*P* = 0.0450).

Finally, a statistically non-significant trend toward decreased NE concentrations from treadmill running was observed compared to the control condition in the remaining cortical areas [*F*(1,32) = 2.87, *P* = 0.0998].

##### 3.2.2.4. Hypothalamic area

No individual neurochemical concentrations were notably influenced by stress or treadmill running conditions (Supplementary Figure 1B). However, prior exposure to stress augmented 5HT turnover measures and further increased the ratio of 5HT:DA turnover in response to treadmill running. Indeed, there was a significant interaction between treadmill running and stress conditions for 5HT turnover measures [*F*(1,32) = 10.69, *P* = 0.0026], without an effect of DA turnover (Figures 5D, E). *Post hoc* analysis showed that the Stress Run group had an augmented ratio of 5HIAA:5HT compared to No Stress Control (*P* = 0.0006), Stress Control (*P* < 0.0001), and No Stress Run groups (*P* = 0.0173). The lack of group differences in DA along with elevated 5HT turnover measures caused an interaction between running and stress conditions for the ratio of 5HT:DA turnover [*F*(1,32) = 5.23, *P* = 0.0290] (Figure 5F). *Post hoc* analysis revealed that the Stress Run group had greater measures of 5HT:DA turnover than No Stress (*P* = 0.0002) and Stress (*P* < 0.0001) Control groups. Moreover, the No Stress Run group had a higher ratio of 5HT:DA turnover than the Stress Control group (*P* = 0.0212).

##### 3.2.2.5. Hippocampal area

**FIGURE 6:**
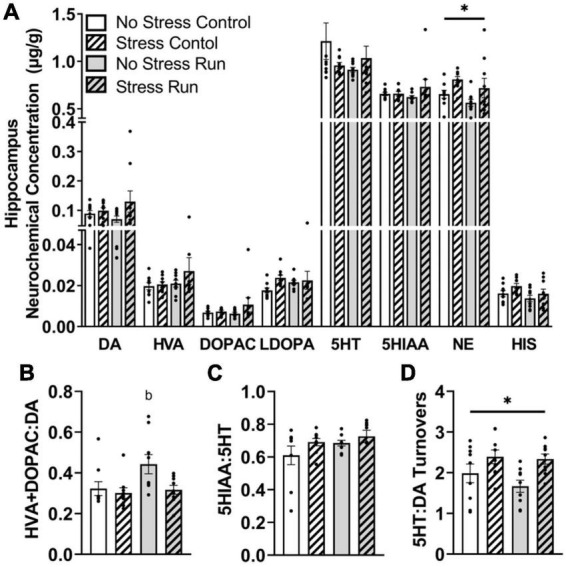
The influence of stress on monoamine-related neurochemical profiles in the hippocampus following a graded bout of treadmill exercise. **(A)** Rats exposed to stress had higher NE concentrations compared to non-stressed rats. **(B)** Stress exposure prevented the treadmill exercise-induced elevation of DA turnover. **(C)** Neither stress nor treadmill exercise influenced 5HT turnover measures. **(D)** Stress exposure overall increased the ratio of 5HT:DA turnover. Bars graphs are group mean (± SEM) with dots representing each animal’s neurochemical concentration. *P < 0.05 main effect of stress. ^b^*P* < 0.05 from Stress Control.

No main effects of treadmill running, stress, or interactions between these factors were observed for individual DA- or 5HT-related neurochemicals. Although a trend toward a stress-induced increase of DA concentration that did not research statistical significance was observed in the hippocampus [*F*(1,32) = 2.90, *P* = 0.0983] (Figure 6A). Despite this, treadmill running increased DA turnover measures [*F*(1,32) = 4.15, *P* = 0.0497], but this effect was lessened by stress exposure [*F*(1,32) = 4.77, *P* = 0.0365] (Figure 6B). Indeed, *post hoc* analysis revealed that the No Stress Run group had a greater ratio of HVA + DOPAC:DA than Stress Control group (*P* = 0.0275), though marginally non-significant trends in the same direction were also observed for No Stress Control (*P* = 0.0782) and Stress Run (*P* = 0.0602) groups. Stress and running did not notably influence 5HT turnover measures (Figure 6C). However, the ratio of 5HT:DA turnover in the hippocampus was augmented by stress [*F*(1,32) = 9.68, *P* = 0.0039] (Figure 6D).

Finally, stress exposure increased NE [*F*(1,32) = 6.62, *P* = 0.0149] and caused a statistically non-significant trend toward increased HIS [*F*(1,32) = 3.15, *P* = 0.0853] concentrations compared to no stress conditions in the hippocampal area.

##### 3.2.2.6. Brainstem

**FIGURE 7:**
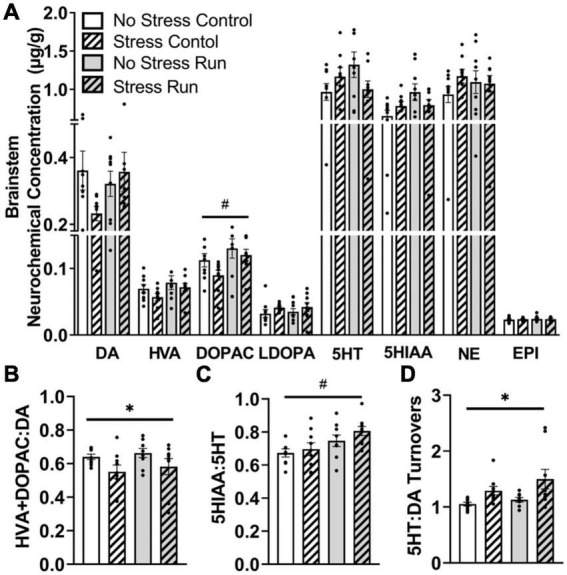
The influence of stress and a bout of treadmill exercise on monoamine-related neurochemical profiles in the brainstem. **(A)** Treadmill running increased DA metabolite DOPAC concentrations in the brainstem. **(B)** Stress exposure lowered DA turnover measures. **(C)** Treadmill running increased 5HT turnover measures in the brainstem. **(D)** Stress exposure overall increased the ratio of 5HT:DA turnover in the brainstem. Bars graphs are group mean (± SEM) with dots representing each animal’s neurochemical concentration. *P < 0.05 main effect of stress, ^#^*P* < 0.05 main effect of exercise.

Treadmill running increased metabolite DOPAC concentrations compared to the control condition in the brainstem area [*F*(1,32) = 6.62, *P* = 0.0149] (Figure 7A). However, statistically non-significant trends for interactions between treadmill running and stress conditions were also observed for DA [*F*(1,32) = 3.14, *P* = 0.0860], 5HT [*F*(1,32) = 4.12, *P* = 0.0508], and 5HIAA [*F*(1,32) = 4.03, *P* = 0.0532].

Stress exposure mildly decreased measures of DA turnover [*F*(1,32) = 5.97, *P* = 0.0202] (Figure 7B), whereas treadmill running increased 5HT turnover measures in the brainstem [*F*(1,32) = 8.29, *P* = 0.0071] (Figure 7C). This contributed to a stress-induced augmentation in the ratio of 5HT:DA turnover measures in the brainstem [*F*(1,32) = 9.35, *P* = 0.0045] (Figure 7D).

##### 3.2.2.7. Cerebellum

Treadmill exercise increased metabolite DOPAC concentration [*F*(1,32) = 4.36, *P* = 0.0449] and displayed a statistically non-significant trend toward increased HVA concentration [*F*(1,32) = 3.29, *P* = 0.0791]. A trend toward a stress-induced reduction in DA turnover measures that did not research statistical significance was also observed in the cerebellum [*F*(1,31) = 3.2.92, *P* = 0.0976]. No significant effects of stress, exercise or interactions were observed for the remaining neurochemical measures (Supplementary Figures 2A–D).

#### 3.2.3. Muscle protein analysis

**FIGURE 8:**
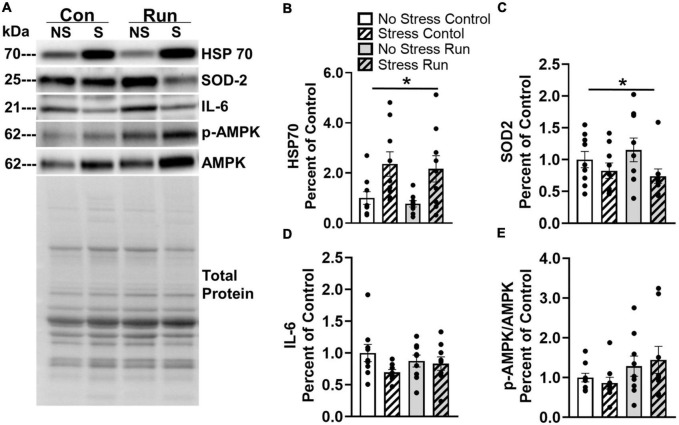
Changes to markers of oxidative stress observed in the hind limb muscle of rats 8-days following stress exposure. **(A)** Representative image of blots for HSP70, SOD 2, IL-6, and p-AMPK/AMPK protein concentrations in the rat gastrocnemius muscle. **(B)** Stress exposure potently increased muscle HSP70 protein concentration. **(C)** Stress also lowered muscle SOD2 protein concentration. **(D)** Stress caused a statistically non-significant trend toward lower muscle IL-6 concentrations. **(E)** A graded bout of treadmill exercise resulted in a statistically non-significant trend toward elevated muscle p-AMPK/AMPK protein concentrations. Bars graphs are group mean (± SEM) with dots representing each animal’s protein expression. Note that representative images and figures for remaining muscle proteins are located in the Supplementary Figures 1–3. *P < 0.05 main effect of stress.

Two proteins in the gastrocnemius muscle were influenced by prior stress exposure (Figure 8A). Rats exposed to stress had increased HSP70 [*F*(1,32) = 12.76, *P* = 0.0011] and decreased SOD2 concentrations [*F*(1,32) = 4.373, *P* = 0.0445] compared to non-stressed rats (Figures 8B, C). A trend that failed to reach statistical significance was observed for a reduction of IL-6 was also observed in stressed rats compared to non-stressed rats [*F*(1,32) = 2.967, *P* = 0.0946] (Figure 8D). No statistically significant main effects of stress or interactions between stress and exercise were observed for any other muscle factors investigated in this study (Supplementary Figure 3).

Rats that ran on treadmills displayed a statistically non-significant trend toward elevated AMPK phosphorylation compared to control rats [*F*(1,32) = 3.53, *P* = 0.0693] (Figure 8E). However, no other notable running effects or trends were observed in for any other muscle factors (Supplementary Figure 3).

## 4. Discussion

**FIGURE 9:**
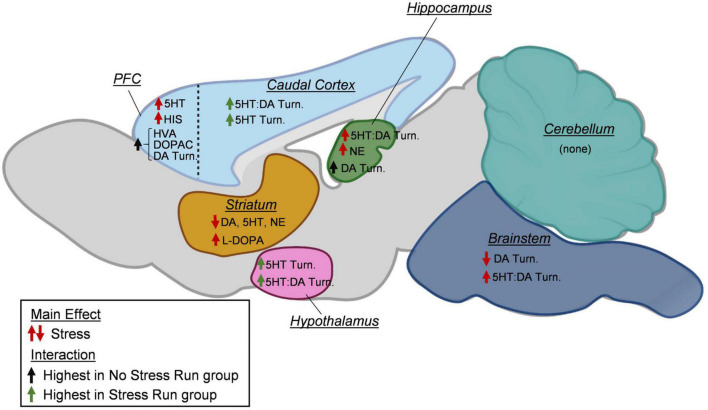
A summary of changes to neurochemical concentrations as a result of stress exposure. In the prefrontal cortex (PFC), prior exposure to stress increased serotonin (5HT) and histamine (HIS) concentrations. Increases in dopamine (DA) turnover measures and DA metabolite concentrations (HVA, DOPAC) during treadmill running were blunted by prior stress exposure. In the caudal cortical areas, exposure to stress increased the turnover of 5HT and the ratio of 5HT:DA turnovers during treadmill running. In the hippocampus, stress increased the ratio of 5HT:DA turnover and norepinephrine (NE). Moreover, increases in dopamine turnover measures during treadmill running were prevented by prior stress exposure. In the striatum, prior exposure to stress lowered DA, 5HT, and NE concentrations, while increasing L-DOPA content. In the hypothalamus, stress increased 5HT turnover and the ratio of 5HT:DA turnover. In the brainstem, stress lowered DA turnover measures and increased the ratio of 5HT:DA turnover. In the cerebellum, no stress effects were observed for any neurochemicals. Created with BioRender.com.

A growing literature suggests that exposure to adverse experiences may become detrimental to the long-term physical activity engagement in humans and rodent models (Moraska and Fleshner, 2001; Stults-Kolehmainen and Sinha, 2014; Vasquez et al., 2016; DeVallance et al., 2017; van den Berk-Clark et al., 2018; Teychenne et al., 2019; Jiang et al., 2020; Raney et al., 2022). However, the biological factors mediating the relationship between acute stress and sedentary lifestyles have yet to be established. The results of this study reveal several novel findings that could have relevance for understanding the relationships between adverse experience and persistent physical activity deficits. First, rat wheel running levels were inversely proportional to the number of tail shocks they received during the episode of stress, suggesting a direct relationship may exist between intensity of stressor and degree of long-term physical activity deficit. Second, a few of the brain monoamine measures that changed in response to a controlled bout of exercise became further altered in rats that were previously exposed to stress, which could have relevance for understanding persistent reductions in physical activity (Figure 9). Stress exposure curbed the exercise-induced augmentation of DA turnover in the prefrontal cortical and hippocampus, as well as heightened 5HT turnover measures in the hypothalamus and remaining cortical area. Third, other stress-induced monoaminergic changes were observed independent of physical activity status that may underlie impaired motivation for physical activity, including a mild deficiency in striatal dopamine levels. Finally, protein concentrations of several factors involved in oxidative stress, inflammation, and energy balance were also assessed in rat hind limb skeletal muscle. Stress exposure potently increased HSP70 and lowered SOD2 protein concentrations in the gastrocnemius muscle, which may be indicative of prolonged oxidative stress. Taken together, these data provide preliminary insight into central and peripheral physiological responses to adverse experiences that may mediate long-term deficits in physical activity output.

Despite our growing awareness of the benefits that regularly engaging in exercise has on both physical health and cognitive function, the proportion of physically inactive individuals remains high, and we appear no closer to a solution for this epidemic (Brownson et al., 2005; Boyington et al., 2015; Piercy, 2019; Yang et al., 2019). Part of the insufficiency of modern approaches to address this issue is that the internal biological processes that regulate the adherence to exercise behavior itself are not well understood (Boyington et al., 2015; Lightfoot et al., 2018). Mounting evidence indicates that exposures to adverse experiences (e.g., psychological traumas) are associated with a host of later-life chronic illnesses that may be mediated, at least, in part by physically inactive lifestyles (Booth et al., 2008; McTiernan, 2008; Chau et al., 2015; Gonzalez et al., 2017; Guthold et al., 2018; Park et al., 2020). In the current study, wheel running deficits were proportional to the number of shocks that the rats were exposed to during stress, which could suggest that a direct relationship may exist between the strength of adverse experience and degree of long-term physical activity deficit. It should be noted that the reported wheel running deficits persisted for 9 weeks in stressed rats without a trend toward recovery to non-stressed running levels. A comprehensive body of research spanning nearly six decades into this exact stress model suggests rats recover from a sequelae of anxiety- and depression-like behaviors in approximately 72 h (Maier and Watkins, 2005; Fleshner et al., 2011; Maier and Seligman, 2016). The relatively short-lived behavioral deficits include motivated processes, such as preference for sweet substances (Christianson et al., 2008) and drugs of abuse (Will et al., 1998), instrumental learning (Maier and Watkins, 2005; Strong et al., 2011), social exploration (Christianson et al., 2008), goal-directed spatial learning (Warren et al., 1991), and food consumption (Desan et al., 1988), as well as exaggerated fear responses (Maier and Watkins, 2005). These data suggest that long-term running deficits are not just a simple correlate of stress-induced depression or anxiety, but instead may be specific to physical exertion. In support of this notion, a recent study also found that mice exposed to 2 h of daily restraint stress over a 2-week period displayed mild wheel running deficits without impairments for sucrose preference compared to non-stressed mice (Scott et al., 2023).

Moreover, behavioral deficits on tasks that require forced episodes of vigorous physical activity also recover to non-stressed levels within the same 72 h period following uncontrollable tail shocks, including 15 min of forced swimming and approximately an hour of shuttle box escape testing (Weiss et al., 1981; Maier and Watkins, 2005; Strong et al., 2011). These data suggest that acutely stressed rats are capable of performing demanding motor activities at high levels when prompted by other motivating factors (e.g., stressors), however, seemingly develop a preference not to engage when physically demanding activities are voluntary. While stress-related deficits of both voluntary wheel (see Figure 1A) and forced treadmill running (see Figure 2B) were observed in the current study, it is possible that the comparatively less aversive approach used in this study to encourage effort during the treadmill task (i.e., more habituation trials, gentle prod with a tongue depressor) made the procedure less compulsory for rats compared to a perceived risk of downing during forced swimming or the fear of foot shocks during shuttle box escape (Weiss et al., 1981; Maier and Watkins, 2005; Strong et al., 2011). In support of this, studies using foot shocks or high-pressure bursts of air to motivate treadmill running typically report longer times to reach failure than the current study, even in relatively exercise-naïve rats (Abdelmalki et al., 1997; Senturk et al., 2001; Davis et al., 2003; Copp et al., 2009; Cordeiro et al., 2014; Zaretsky et al., 2018; Hou et al., 2019; Rabelo et al., 2019). Moreover, rats that were habituated to the treadmill apparatus fewer times before testing also did not display deficits in running duration in the current study (see Figure 2A). Taken together, persistent running deficits caused by uncontrollable tail shock may not only serve as a useful approach for understanding the biological factors mediating exposure to adverse events and the development of physically inactive lifestyles, but also the physiological underpinnings of exercise motivation.

The biological mechanisms underlying differences in physical activity engagement remain poorly understood. However, outcomes of human and rodent research on differences in exercise motivation continue to draw attention to variations in monoamine signaling in brain reward, motor, and limbic circuits. Differences in rodent spontaneous wheel running levels have been attributed to changes in brain 5HT and DA activities, genes that regulate monoamine signaling (e.g., NHLH2, dopamine receptors Drd1 and Drd2), and transcriptional accessibility of 5HT receptor genes (Rhodes and Garland, 2003; Good et al., 2015; Claghorn et al., 2016; Saul et al., 2017; Greenwood, 2019; Tanner et al., 2022). Moreover, declines in exercise performance are correlated with reductions in DA, elevations in 5HT, and bidirectional changes to NE activity across brain reward, motor, and limbic systems (Meeusen et al., 2006). Given the converging evidence for monoamine involvement in the regulation of physical activity levels, as well as the profound abnormalities in DA and 5HT activity that have been characterized during the 72 h period following uncontrollable tail shock exposure (Amat et al., 1998a,b; Bland et al., 2003; Maier and Watkins, 2005; Greenwood et al., 2012; Clark et al., 2015), perhaps stress of sufficient intensity could persistently alter monoaminergic responses to bouts of exercise that, in turn, impair exercise output (Greenwood, 2019). In support of this hypothesis, data from the current study suggest that prior stress exposure blunted the exercise-induced increase of DA turnover in the prefrontal cortex and the hippocampus, while also augmenting 5HT turnover in the hypothalamus and caudal cortical area. Very few definitive studies have investigated the involvement of brain regional monoamine activity in exercise motivation. However, some evidence supporting the region-specific involvement of DA and 5HT in effortful behavior and stress hormone regulation might shed light on the possible consequences of the stress-induced changes to these neurotransmitters’ turnover in response to bouts of exercise. For instance, the prefrontal cortex has been suggested to play a key role in effort perception during exercise (Berchicci et al., 2013), where increased DA transmission may contribute to the maintenance of motivated behaviors when high levels of effort are required (Floresco and Ghods-Sharifi, 2007; Kurniawan et al., 2011; Treadway et al., 2012). Therefore, the capacity of stress exposure to prevent an exercise-induced enhancement of DA transmission in the prefrontal cortex may contribute to a reduced motivation to engage in physically exertive behaviors. Moreover, a positive relationship exists between hypothalamic 5HT activity and the release of stress hormones (Hanley and Van de Kar, 2003; Heisler et al., 2007), suggesting that prior exposure to tail shock may evoke an even stronger stress response to bouts exercise (Brownlee et al., 2005; Budde et al., 2015), which could chronically deter physical activity engagement. However, future studies are needed to establish how these stress-induced changes in monoaminergic responses to exercise might be causally linked to deficits in physical activity engagement.

In addition, observed changes to monoamine neurochemical concentrations that occurred from stress independent from interactions with running could also have implications for persistent physical activity deficits. Prior stress exposure augmented HIS and 5HT in the prefrontal cortex, as well as NE in the hippocampus (Figure 9). Stress also resulted in a lower DA, 5HT, and NE content within the striatum, as well as DA turnover in the brainstem. While many of these changes could interact in complex manners to influence exercise behavior, a hypodopaminergic state in striatum might be particularly relevant for understanding running motivation deficits (Greenwood, 2019). Dopamine 1 (D1) and 2 (D2) receptors have relatively high degrees of expression and predominate post-synaptic locations of striatal medium spiny neurons, where they have been shown to be major modulators of striatum function. Stimulation of striatal D1 and D2 receptors increases spontaneous running behavior, whereas blockade of these receptors has the opposite effect (Rhodes and Garland, 2003; Knab et al., 2012; Beeler et al., 2016; Ebada et al., 2016; Zhu et al., 2016). Therefore, a stress-induced deficiency in striatal DA concentrations could lead to reduced activity at D1 and D2 receptors, thereby persistently lowering physical activity output.

However, it is also worth highlighting research suggesting dopamine deficiencies in the striatum do not just impair spontaneous physical activities, but also disrupt a multitude motivated behaviors including preference for sweet substances, goal-directed learning, social interactions, and feeding behaviors (Szczypka et al., 2001; Palmiter, 2008; Rincon-Cortes and Grace, 2021). Deficits in these motivated behaviors have been well-characterized in the uncontrollable tail shock model and recover before the 8 days post-stress period that striatal DA deficiencies were observed in this study (Desan et al., 1988; Warren et al., 1991; Maier and Watkins, 2005; Christianson et al., 2008; Strong et al., 2011). Therefore, how striatal DA deficits might contribute to lasting changes in physical activity without disrupting other motivated behaviors is not entirely clear. One possibility is that voluntary behaviors that require a greater degree of exertion may be more sensitive to relatively mild reductions in DA, like observed in this study. Whereas, more potent dopamine deficiencies may be necessary to impair other motivated behaviors that do not require as much effort, like preferences for sweet substances or social interaction. Indeed, studies reporting a wide array of deficits in motivated behaviors often employ transgenic approaches or DA neuron lesions that greatly reduce striatal DA content (Szczypka et al., 2001; Palmiter, 2008). Moreover, exercise engagement may be susceptible to subtle variations of DA levels within the ventral striatum in particular, as this region has been shown to mediate the hedonic tone during effort-based behaviors, including exertion during physical activities (Barraco et al., 1993; Salamone et al., 2007; Zhu et al., 2016; Bryce and Floresco, 2019). The exact mechanisms causing lasting striatal DA deficits following adverse events, as well as its possible involvement in specifically impairing physically exerting voluntary behaviors warrant further mechanistic investigation, as it could provide insight into how exposure to adverse events can chronically impair physical activity engagement.

Skeletal muscle physiology continues to remain a focus of research on variations in exercise motivation and output. Studies have demonstrated that the overexpression of glucose transporters or knockdown of immune factors in muscle can markedly increase spontaneous running activity in mice (Tsao et al., 2001; Pistilli et al., 2007). Skeletal muscle genetic and proteomic factors that regulate differences in physical activity levels remain key areas of interest in understanding exercise motivation (Lightfoot et al., 2018). In the current study, a lack of statistical significance in the many biomarkers for energy balance, oxidative stress, and inflammation does not preclude their possible involvement in stress-induced running deficits, but instead is likely a limitation of the single post-running sample point. For instance, immune factor changes can vary in magnitude and direction from hours to days post-exercise (Weinstock et al., 1997; Peake et al., 2017). Yet still, prior tail shock exposure increased HSP70 expression, a protein involved in cytoprotection in a response to cellular stress (Tupling, 2008; Li et al., 2011), as well as lowered SOD2 levels, an antioxidant involved in maintaining redox conditions in mitochondria (Franco et al., 1999). These two observations are intriguing with respect to impairing physical activity levels in stress-exposed rats. HSP70 can become overexpressed as a part of the cellular response to oxidative stress and serves as a chaperone to stabilize the appropriate folding of proteins (Tupling, 2008). Therefore, the overexpression of HSP70 may indicate increased oxidative stress in the muscle cells, which, if not contained, can lead to apoptosis (Mosser et al., 1997; Ahn et al., 1999). Over time, this could lead to necrosis of healthy muscle mass and impaired exercise performance.

The stress-related striking increase of HSP70, as well as the reduction in SOD2, may be further suggestive of a reduction in the antioxidant capacity of skeletal muscle with a possible outcome of greater oxidative stress. In fact, the reduction in SOD2 may be partly responsible for fatigue originating in skeletal muscle during exercise, which could contributes to earlier failure on the treadmill test observed in stressed rats (see Figure 2B). Indeed, it is well-established that the accumulation of free radicals in skeletal muscle contribute to muscular fatigue (Powers et al., 2016). As radicals form due to increased mitochondrial flux during exercise (Davies et al., 1982), the lack of SOD2 availability may lower the antioxidative capacity of muscle in stressed rats (Lustgarten et al., 2009). The shift from an optimal redox balance to one dominated by free radical formation in the muscle of stressed rats could impair force production and hasten fatigue onset (Lustgarten et al., 2009; Powers et al., 2016). Indeed, SOD2 knockdown in mouse gastrocnemius muscle induces potent deficits in daily wheel running distances (Lustgarten et al., 2009). Therefore, HSP70 levels may be indicative of increased oxidative stress that could result, in part, from a lower antioxidant capacity due to reduced SOD2 in muscle. How long these changes in skeletal muscle exist following acute stress could provide useful information, given that running deficits persist for at least months. The potential influence of persistently diminished antioxidant capacity and greater oxidative stress on skeletal muscle contraction, and its possible role in long-term impairments to exercise output following exposure to adverse experiences requires further study.

## 5. Conclusion

In conclusion, the current study provides some insight into the central and peripheral mechanisms by which exposures to adverse experiences lead to persistently impaired physical activity engagement. Not only did prior stress exposure result in several changes to monoamine patterns throughout the brain that could influence physical exertion including striatal dopamine deficiencies, but it also augmented 5HT turnover measures in response to exercise within the hypothalamus and caudal cortex, as well as prevented running-increased DA turnover in the prefrontal cortex and hippocampus (Figure 9). Moreover, elevated HSP70 and reduced SOD2 are consistent with a stress-induced increase of oxidative stress in hind limb muscle that could contribute to impaired physical activity levels. The potential relationships between stress-induced changes to skeletal muscle physiology and brain factors that regulate neurochemical responses to exercise could be important topics of future investigations. Moreover, future studies are needed to build upon these data to understand how stress-induced changes to monoaminergic response might interact across brain regions in manners that habitually disrupt physical activity levels. Finally, given the range of running distances observed across outbred rats in each group (see Supplementary Table 1), the biological factors mediating individual variation in response to stress (e.g., stress susceptibility) could also provide a future direction for research (Chaudhury et al., 2013; Hollis et al., 2015; Zalachoras et al., 2022). Identifying persistent stress-induced disturbances to brain and muscle physiology that impair rat running behavior may further our understanding of the internal processes that leave individuals more prone to physical inactivity across the lifespan.

## Data availability statement

The raw data supporting the conclusions of this article will be made available by the authors, without undue reservation.

## Ethics statement

This animal study was reviewed and approved by the Iowa State University Institutional Animal Care and Use Committee.

## Author contributions

TB and CR contributed to the conception, design, data analysis, and writing of the manuscript. OW and JL contributed to the data collection and analysis. L-LY contributed to the data analysis and writing the manuscript. MF funded the studies and contributed to the data analysis and writing of experiment 1. RV contributed to the conceptualization, funding, data interpretation, and writing of the muscle sections. PC contributed to the funding, conception, design, data analysis, interpretation, and writing and final editing of the manuscript. All authors contributed to the article and approved the submitted version.
