# Supplementary figures and images for: The persistence of stress-induced physical inactivity in rats: an investigation of central monoamine neurotransmitters and skeletal muscle oxidative stress
Source: Front Behav Neurosci. 2023 May 16;17:1169151. doi: 10.3389/fnbeh.2023.1169151 (PMC10237271; doi:10.3389/fnbeh.2023.1169151)

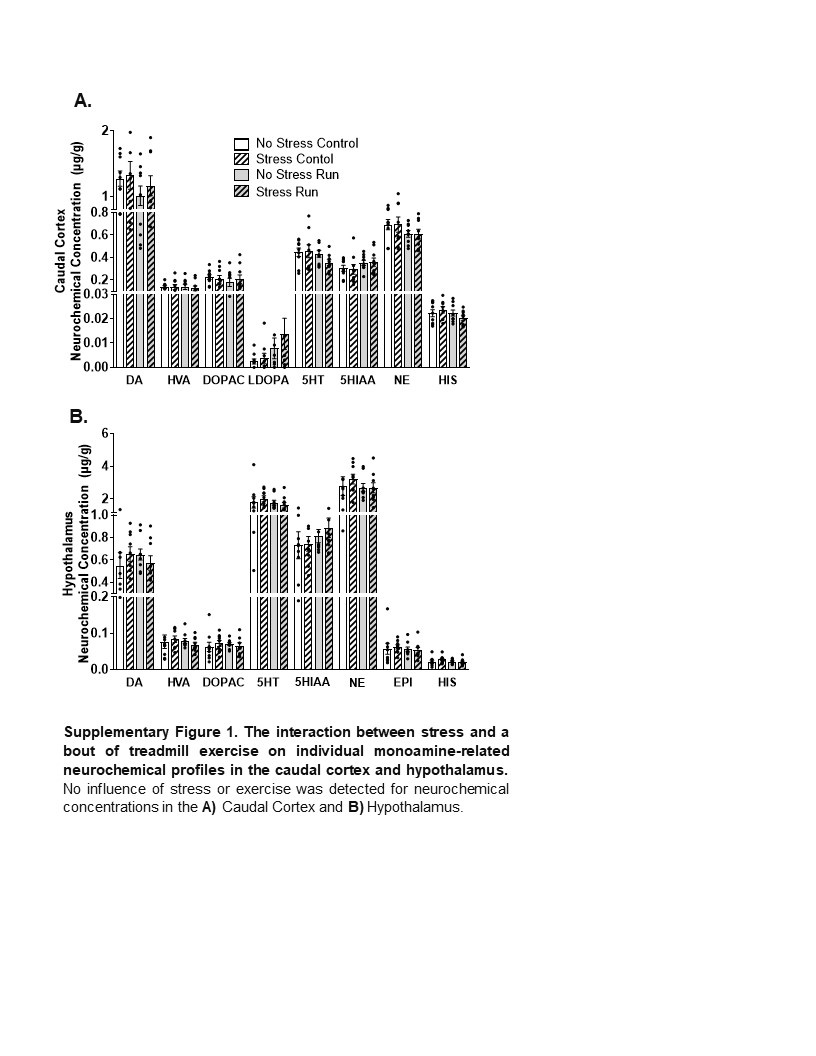

Supplement: Supplementary file 1 [file Image_1.jpeg]

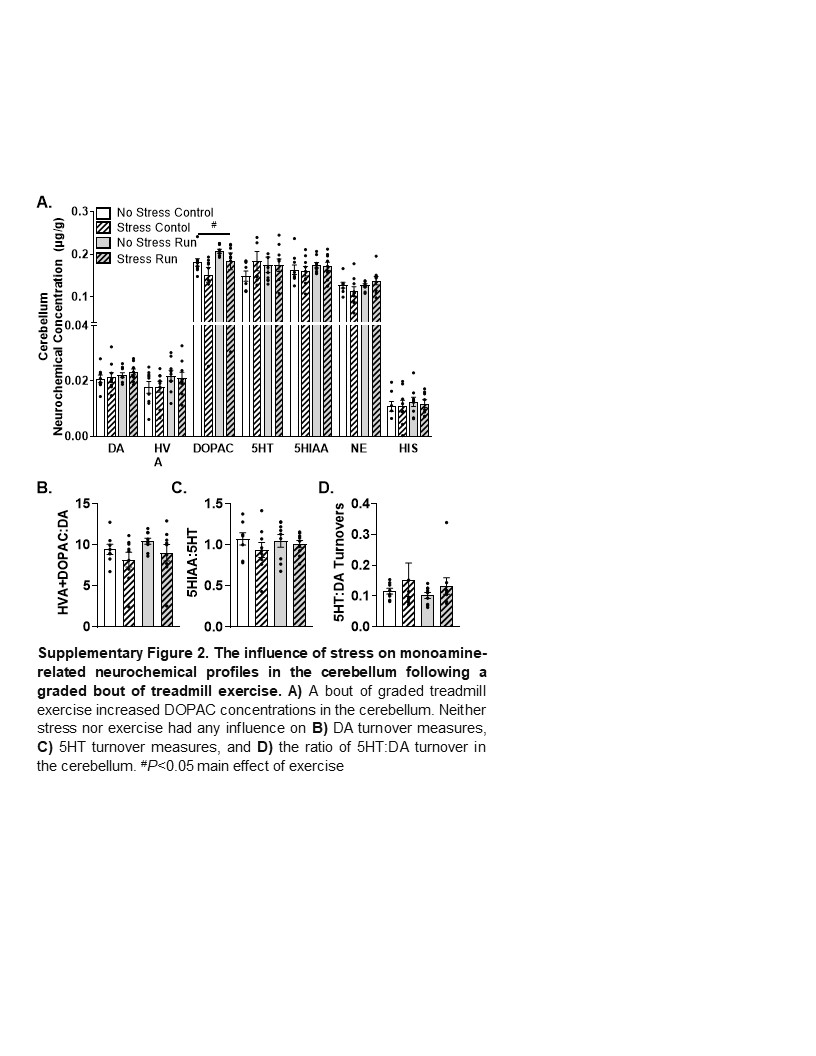

Supplement: Supplementary file 2 [file Image_2.jpeg]

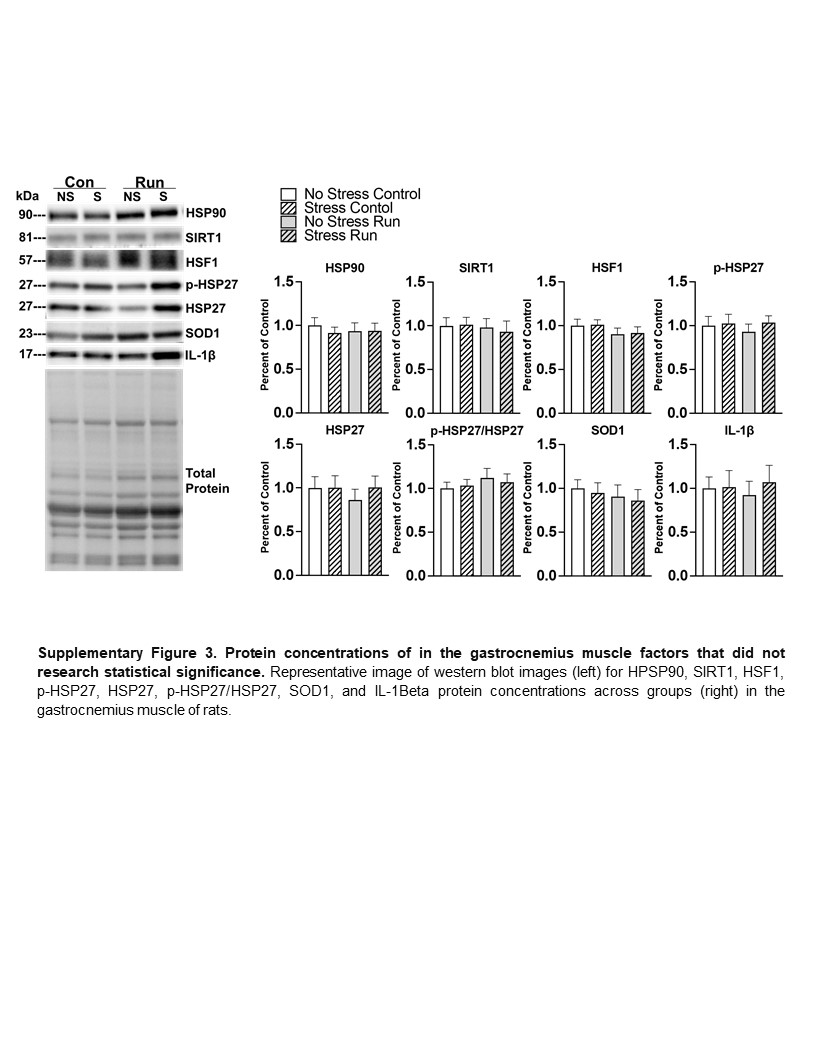

Supplement: Supplementary file 3 [file Image_3.jpeg]
