# Supplementary material for: The persistence of stress-induced physical inactivity in rats: an investigation of central monoamine neurotransmitters and skeletal muscle oxidative stress
Source: Front Behav Neurosci. 2023 May 16;17:1169151. doi: 10.3389/fnbeh.2023.1169151 (PMC10237271; doi:10.3389/fnbeh.2023.1169151)
Supplement: Supplementary file 4 [file Table_1.docx]

**Supplementary Table 1.** Average weekly running distance for rats over the entire duration of the experiment.

| **Animal Number** | **Stress Condition** | **Ave. Distance**  **Run (Km)** | **Std. Error** |
| --- | --- | --- | --- |
| 1 | 0 Shocks | 1.83 | 0.09 |
| 2 | 0 Shocks | 3.38 | 0.76 |
| 3 | 0 Shocks | 4.72 | 0.80 |
| 4 | 0 Shocks | 9.01 | 2.29 |
| 5 | 0 Shocks | 5.70 | 0.89 |
| 6 | 0 Shocks | 3.69 | 0.44 |
| 7 | 50 Shocks | 2.08 | 0.35 |
| 8 | 50 Shocks | 2.37 | 0.25 |
| 9 | 50 Shocks | 6.83 | 1.62 |
| 10 | 50 Shocks | 4.70 | 1.11 |
| 11 | 50 Shocks | 0.94 | 0.11 |
| 12 | 50 Shocks | 1.78 | 0.29 |
| 13 | 100 Shocks | 1.84 | 0.37 |
| 14 | 100 Shocks | 1.18 | 0.11 |
| 15 | 100 Shocks | 2.14 | 0.31 |
| 16 | 100 Shocks | 0.79 | 0.07 |
| 17 | 100 Shocks | 2.93 | 0.79 |
| 18 | 100 Shocks | 1.90 | 0.25 |
